# Exploring the Use of YouTube as a Pathology Learning Tool and Its Relationship With Pathology Scores Among Medical Students: Cross-Sectional Study

**DOI:** 10.2196/45372

**Published:** 2023-11-24

**Authors:** Hiba Alzoubi, Reema Karasneh, Sara Irshaidat, Yussuf Abuelhaija, Saleh Abuorouq, Haya Omeish, Shrouq Daromar, Naheda Makhadmeh, Mohammad Alqudah, Mohammad T Abuawwad, Mohammad J J Taha, Ansam Baniamer, Hashem Abu Serhan

**Affiliations:** 1 Department of Basic Medical Sciences Faculty of Medicine Yarmouk University Irbid Jordan; 2 Department of Pediatric King Hussein Cancer Center Amman Jordan; 3 Department of Clinical Medicine Gardens Hospital Amman Jordan; 4 Urology Division, Department of Clinical Medical Sciences Faculty of Medicine Yarmouk University Irbid Jordan; 5 King Hussein Medical Centre Amman Jordan; 6 Department of Pathology and Microbiology The University of Jordan Amman Jordan; 7 Department of Journalism College of Mass Communication Yarmouk University Irbid Jordan; 8 Pathology Division, Department of Basic Medical Sciences Faculty of Medicine Jordan University of Science and Technology Irbid Jordan; 9 Department of Clinical Medicine Kasr Alainy Faculty of Medicine Cairo University Cairo Egypt; 10 Faculty of Medicine Yarmouk University Irbid Jordan; 11 Department of Ophthalmology Hamad Medical Corporations Doha Qatar

**Keywords:** pathology, medical students, YouTube, social media, medical education, online resources

## Abstract

**Background:**

YouTube is considered one of the most popular sources of information among college students.

**Objective:**

This study aimed to explore the use of YouTube as a pathology learning tool and its relationship with pathology scores among medical students at Jordanian public universities.

**Methods:**

This cross-sectional, questionnaire-based study included second-year to sixth-year medical students from 6 schools of medicine in Jordan. The questionnaire was distributed among the students using social platforms over a period of 2 months extending from August 2022 to October 2022. The questionnaire included 6 attributes. The first section collected demographic data, and the second section investigated the general use of YouTube and recorded material. The remaining 4 sections targeted the participants who used YouTube to learn pathology including using YouTube for pathology-related content.

**Results:**

As of October 2022, 699 students were enrolled in the study. More than 60% (422/699, 60.4%) of the participants were women, and approximately 50% (354/699, 50.6%) were second-year students. The results showed that 96.5% (675/699) of medical students in Jordan were using YouTube in general and 89.1% (623/699) were using it as a source of general information. YouTube use was associated with good and very good scores among the users. In addition, 82.3% (575/699) of medical students in Jordan used YouTube as a learning tool for pathology in particular. These students achieved high scores, with 428 of 699 (61.2%) students scoring above 70%. Most participants (484/699, 69.2%) reported that lectures on YouTube were more interesting than classic teaching and the lectures could enhance the quality of learning (533/699, 76.3%). Studying via YouTube videos was associated with higher odds (odds ratio [OR] 3.86, 95% CI 1.33-11.18) and lower odds (OR 0.27, 95% CI 0.09-0.8) of achieving higher scores in the central nervous system and peripheral nervous system courses, respectively. Watching pathology lectures on YouTube was related to a better chance of attaining higher scores (OR 1.96, 95% CI 1.08-3.57). Surprisingly, spending more time watching pathology videos on YouTube while studying for examinations corresponded with lower performance, with an OR of 0.46 (95% CI 0.26-0.82).

**Conclusions:**

YouTube may play a role in enhancing pathology learning, and aiding in understanding, memorization, recalling information, and obtaining higher scores. Many medical students in Jordan have positive attitudes toward using YouTube as a supplementary pathology learning tool. Based on this, it is recommended that pathology instructors should explore the use of YouTube and other emerging educational tools as potential supplementary learning resources.

## Introduction

YouTube, which launched in 2005, gained tremendous progressive popularity as an online video-sharing website, and it remains the most popular social media platform in the world, with more than 8.5 billion monthly visitors. In addition, YouTube.com is the most visited website. Over 2 billion users log in to YouTube each month, and the users consume over 1 billion hours of content daily [1].

Although YouTube was invented as a video-sharing platform for the everyday user, the tendency for educational use has not gone unnoticed. Over time, scores of colleges and universities have established a presence and created their own tips on YouTube by using their own video-sharing web pages called YouTube channels. In March 2009, YouTube announced the launch of YouTube EDU, which was a highly organized collection of YouTube channels synthesized by college and university partners. At the end of YouTube EDU’s first year, YouTube EDU had grown to include more than 300 colleges and universities with more than 65,000 videos of lectures, news, and campus life available freely for the public view [2].

Currently, YouTube is considered one of the most popular sources of information among college students. In addition to YouTube videos providing students with immediate information freely, using YouTube videos is noted to improve students’ engagement, increase their perception of learning efficacy, enhance and sharpen critical thinking, and aid their deep understanding and visualization of the viewed materials [3,4]. However, traditional teaching using boards, laptop screens, and projectors is progressively declining and becoming more boring to many students. This is becoming obvious, especially with the emergence of different highly interactive technology-based educational and learning tools [5]. Several studies postulated that an effective educational role could be played by technology-based tools such as social media websites including YouTube [6].

Pathology is the study of disease, and it is the bridge between basic and clinical sciences for medical students. It concerns every aspect of patient care, from diagnosis to treatment, and it is an essential science for the clinical practice of future doctors. Given that pathology material is full of gross and microscopic images, it is expected that learning pathology requires more visual methods like YouTube videos and other attractive technology methods that increase visual engagement to enhance learning. A continuously increasing number of pathology-related videos are available on YouTube. These videos include many recorded lectures, animations, microscopic and gross illustrations, tutorials for gross dissection of anatomical organs, and much more. As previously noted, with a well-organized video, anatomical footage of live patients along with cadavers, medical imaging, plastic models, and diagrams can be used to enhance and solidify the understanding of 3D structures [7]. Additionally, a UK case study about the use of YouTube videos in learning and teaching an introductory course concluded that the use of YouTube was an effective method of supporting learning and surmised that YouTube helps students learn by providing alternative views and opinions on subjects, a variety of delivery mechanisms, and the use of everyday examples to illustrate points [8]. Such reports suggest that the efficient use of YouTube videos as a student learning aid might also be very helpful for building pathological knowledge that is very important for clinical practice.

This study was designed to confirm that medical students in Jordan recognize YouTube as an important medical and, in particular, pathology educational tool. Consequently, this could positively affect student scores in the pathology course Moreover, we report some challenges faced by students while using YouTube and suggest some recommendations to adopt YouTube as an educational tool in the pathology curricula of universities.

## Methods

### Study Design and Participants

This descriptive, cross-sectional study was conducted using an online questionnaire targeting second-year to sixth-year medical students in all public universities in Jordan. We followed the Checklist for Reporting Results of Internet E-Surveys (CHERRIES) in conducting this study [9]. Data were collected using web-based questionnaire software (Google Forms). Participants were recruited from August 2022 to October 2022 through social media (Facebook) by posting an invitation to complete the questionnaire after providing a brief description of the study. The link to the questionnaire was included in the invitations that were posted on pages and groups commonly accessed by medical students in Jordanian public universities (eg, university-related pages, study group pages).

### Items in the Questionnaire

We used a 10-minute online questionnaire created in Google Forms that was based on current scientific literature by referring to the literature and included main ideas and primary items relevant to our topic. The face and content validity of the questionnaire were established by review of the questionnaire by 5 experts in the field who provided their feedback and suggested necessary changes. The reliability of the questionnaire was established through pilot testing by collecting data from 20 medical students who were asked to provide their feedback. These students were not included in the study sample.

The questionnaire consisted of 6 attributes. The first section collected participant demographic data, including gender, university, and level of study. The second section included 4 questions on the general use of YouTube and recorded material. The remaining 4 sections targeted the participants who used YouTube to learn pathology including using YouTube for pathology-related content. The second section included a comparison of content and presentation between recorded pathology material (lectures or videos) provided by the faculty of medicine at the student’s university and pathology-related YouTube videos viewed by the participant. The comparison used ratings of excellent, good, acceptable, and poor, with an option to choose “not applicable.” The third section assessed the prevalence of the use of YouTube for pathology-related content and learning pathological sciences and systems with “YES” and “NO” answers available. The fourth section included 12 questions on perceived value, challenges, and recommendations for using YouTube as a learning tool for pathology. Students were also asked about time spent watching YouTube videos when preparing for pathology-related examinations and reasons for using YouTube as a learning tool for pathology.

### Sample Size and Sampling Technique

The sample size was calculated using epiinfoTM v.7.2.4.0 (US Centers for Disease Control and Prevention) [10], a database and statistics program for public health professionals, considering a previous study performed in Jordan with a sample population proportion of 68% [11]. Using a cross-sectional study design, where n is the required sample size (n=Z (α/2) 2 pq/d2), we calculated the sample size based on the following parameters: prevalence of 68%, precision of 0.04, 99% CI, and 5% margin of error. We estimated 577 as the minimum sample size required to represent the true population. A convenience sampling size (n=699) was used in this study.

### Data Analysis

Frequencies and proportions were used to summarize the data. Comparisons between categorical variables were analyzed using the Pearson chi-square test. In order to examine the factors linked to students’ pathology exam scores in terms of YouTube utilization, binary logistic regression analysis was performed. Scores above 70% were considered a sufficient level of proficiency in pathology (true), and scores lower than 70% were considered an insufficient level of proficiency in pathology (false). Statistical significance was set at *P*=.05. SPSS version 26 (IBM Corp) was used to conduct the statistical analysis.

### Ethics Approval

Ethics approval was obtained from the Yarmouk University institutional review board (Ref R D\119\12\3460). All study procedures were conducted according to the principles of the World Medical Association Declaration of Helsinki and its amendments [12]. Participants were informed prior to starting the questionnaire that it was completely anonymous and voluntary and that all data would be treated as confidential.

## Results

As of October 2022, a total of 699 Jordanian medical students responded and completed the survey. Of the 699 responses, 445 (63.7%) were collected in Arabic, while the rest were in English. Over 60% (422/699, 60.4%) of the participants were women, and approximately 50% (354/699, 50.6%) of the participants were second-year students. A very good score (81%-90%) in the pathology course was the most often reported score (209/699, 29.9%), followed by 202 good scores (71%-80%). Table 1 describes the demographic characteristics of the participating medical students.

In terms of using YouTube for learning and its relationship with pathology scores, a large proportion of students included in this study (675/699, 96.5%; *P*=.31) acknowledged using it. Similarly, among students who used YouTube in general, very good and good scores were the most frequently described (405/699, 57.9%). Fewer students (623/699, 89.1%) reported using YouTube as a source of general information, with students with very good and good scores being the most frequent to answer this way. Among students who did not use YouTube as a source of learning, using recorded material (lectures or videos) provided by the faculty of medicine at their universities was the least often mentioned source of information (198/699, 28.3%). However, among the students who used the recorded material (lectures or videos) provided by the faculty of medicine, better scores were reported, with very good and good being the most commonly reported scores. When asked if they used YouTube as a learning tool for pathology, the majority of the participants (575/699, 82.3%) responded affirmatively. A comparison of the 2 results (affirmative and negative) revealed that the scores of the students who used YouTube as a source of pathology learning tool were higher, with 428 students scoring above 70%, particularly in comparison with only 90 students scoring the same result but without the use of a YouTube learning source. The detailed results are shown in Table 2.

Concerning the content of the recorded lectures or videos offered by their universities, 233 students rated it as good, a similar number (n=199) stated that it was acceptable, and a slightly smaller number (n=145) described it as poor. Further, students reported comparable results on the presentation quality of the recorded material for the pathology course (lectures or videos) provided by the college of medicine, with approximately 56% (395/699, 56.5%) reporting that the presentation quality was acceptable or good. However, when asked about the pathology-related YouTube videos, 376 and 424 students evaluated them as “excellent” in content and presentation, respectively. Table 3 shows the perceived value of using university pathology lectures compared with YouTube lectures.

When comparing the attitudes of students toward university pathology lectures with their attitudes toward YouTube lectures, most participants found that YouTube lectures are more interesting (484/699, 69.2%), contain a lot of animations and pictures (537/699, 76.8%), and could enhance the quality of learning (533/699, 76.2%). In addition, 68% (475/699) of students answered with “yes” when asked if university pathology lectures are not attractive to all students. Table 4 shows the students' attitudes toward university pathology lectures and YouTube lectures.

In terms of the association between the actual use of YouTube to study pathology and students' grades, 323 students said they had been confused when choosing the appropriate pathology information. However, of the 323 students, 193 received good or very good scores, while a few (n=8) received only a 50% score. Only 99 students, on the other hand, claimed that they had not been confused when selecting the appropriate pathologic information. Most of the students (524/699, 75%) achieved a score higher than 70%. Furthermore, when asked if it would be helpful if your instructor chose appropriate pathological YouTube videos in his lectures, most students who had an overall score in the pathology course >70% agreed. Table 5 summarizes the student opinions on the effectiveness of using YouTube in combination with instructor lectures.

Binary logistic regression analysis was used to correlate the factors that influence students’ scores. These factors were divided into 3 questions: the first regarding the studied module, the second regarding the type of content, and the third question regarding the time spent watching YouTube videos while preparing for pathology exams. Overall, the majority of these variables were indeterministic and statistically insignificant. Nevertheless, studying the central nervous system via YouTube videos was associated with higher odds of achieving higher scores (odds ratio [OR] 3.86, 95% CI 1.33-11.18; *P*=.01). YouTube-assisted study of the peripheral nervous system, on the contrary, was related to lower odds of achieving higher scores (OR 0.27, 95% CI 0.09-0.8; *P*=.02). Similarly, watching pathology lectures on YouTube was related to a better chance of attaining higher scores (OR 1.96, 95% CI 1.08-3.57; *P*=.03), but watching study tips and pieces of advice were correlated with a decreased likelihood of achieving higher scores (OR 0.47, 95% CI 0.29-0.77; *P*=.01). Surprisingly, spending more time watching pathology videos on YouTube while studying for examinations corresponded with lower performance, with an OR of 0.46 (95% CI 0.26-0.82; *P*=.01). Table 6 shows the binary logistic regression analysis results for the factors that contributed to students’ exam performance.

**Table 1:** Demographic characteristics of the participating medical students (n=699).

Characteristic		Total sample, n (%)	Score in the pathology course, n (%)								*P* value	
			<50% (n=25)	50%-60% (n=58)	61%-70% (n=93)	71%-80% (n=202)	81%-90% (n=209)	>90% (n=107)	N/A^a^ (n=5)			
**In which language would you prefer to complete this survey?**												.01
	Arabic	445 (63.7)	3 (0.4)	29 (4.2)	72 (10.3)	142 (20.3)	141 (20.2)	56 (8)	2 (0.3)			
	English	254 (36.3)	22 (3.2)	29 (4.2)	21 (3)	60 (8.6)	68 (9.7)	51 (7.3)	3 (0.4)			
**Sex**												.45
	Female	422 (60.4)	13 (1.9)	41 (5.9)	59 (8.4)	119 (17)	129 (18.5)	58 (8.3)	3 (0.4)			
	Male	277 (39.6)	12 (1.7)	17 (2.4)	34 (4.9)	83 (11.9)	80 (11.4)	49 (7)	2 (0.3)			
**Age** **(years)**												.03
	≤20	391 (55.9)	10 (1.4)	37 (5.3)	55 (7.9)	122 (17.5)	111 (15.9)	53 (7.6)	3 (0.4)			
	21-24	300 (42.9)	13 (1.9)	19 (2.7)	38 (5.4)	78 (11.2)	97 (13.9)	53 (7.6)	2 (0.3)			
	>24	8 (1.1)	2 (0.3)	2 (0.3)	0	2 (0.3)	1 (0.1)	1 (0.1)	0			
**Level of** **study**												<.001
	First year	2 (0.3)	0	0	0	0	1 (0.1)	0	1 (0.1)			
	Second year	354 (50.6)	9 (1.3)	33 (4.7)	49 (7)	115 (16.5)	100 (14.3)	46 (6.6)	2 (0.3)			
	Third year	136 (19.5)	2 (0.3)	12 (1.7)	19 (2.7)	34 (4.9)	48 (6.9)	21 (3)	0			
	Fourth year	84 (12)	4 (0.6)	5 (0.7)	11 (1.6)	25 (3.6)	21 (3)	17 (2.4)	1 (0.1)			
	Fifth year	56 (8)	5 (0.7)	3 (0.4)	9 (1.3)	10 (1.4)	17 (2.4)	11 (1.6)	1 (0.1)			
	Sixth year	67 (9.6)	5 (0.7)	5 (0.7)	5 (0.7)	18 (2.6)	22 (3.2)	12 (1.7)	0			
**University**												.01
	BAU^b^	47 (6.7)	0	9 (1.3)	11 (1.6)	13 (1.9)	11 (1.6)	3 (0.4)	0			
	HU^c^	36 (5.2)	2 (0.3)	9 (1.3)	4 (0.6)	9 (1.3)	6 (0.9)	6 (0.9)	0			
	JUST^d^	167 (23.9)	14 (2)	9 (1.3)	24 (3.4)	46 (6.6)	49 (7)	24 (3.4)	1 (0.1)			
	MU^e^	44 (6.3)	1 (0.1)	11 (1.6)	9 (1.3)	17 (2.4)	3 (0.4)	1 (0.1)	2 (0.3)			
	UJ^f^	85 (12.2)	4 (0.6)	4 (0.6)	10 (1.4)	22 (3.2)	26 (3.7)	18 (2.6)	1 (0.1)			
	YU^g^	320 (45.8)	4 (0.6)	16 (2.3)	35 (5)	95 (13.6)	114 (16.3)	55 (7.9)	1 (0.1)			

^a^N/A: not available.

^b^BAU: Balqa Applied University.

^c^HU: Hashmaite University.

^d^JUST: Jordan University of Science and Technology.

^e^MU: Mutah University.

^f^UJ: University of Jordan.

^g^YU: Yarmouk University.

**Table 2:** The use of YouTube as a learning tool (n=699).

Question		Total sample, n (%)	Score in the pathology course, n (%)								*P* value	
			<50% (n=25)	50%-60% (n=58)	61%-70% (n=93)	71%-80% (n=202)	81%-90% (n=209)	>90% (n=107)	N/A^a^ (n=5)			
**Do you use YouTube in** **general** **?**												.31
	No	24 (3.4)	2 (0.3)	2 (0.3)	4 (0.6)	3 (0.4)	11 (1.6)	2 (0.3)	0			
	Yes	675 (96.6)	23 (3.3)	56 (8)	89 (12.7)	199 (28.5)	198 (28.3)	105 (15)	5 (0.7)			
**Do you use YouTube as a source for general information?**												.36
	No	76 (10.9)	2 (0.3)	5 (0.7)	10 (1.4)	24 (3.4)	25 (3.6)	8 (1.1)	2 (0.3)			
	Yes	623 (89.1)	23 (3.3)	53 (7.6)	83 (11.9)	178 (25.5)	184 (26.3)	99 (14.2)	3 (0.4)			
**Do you use YouTube as a learning tool in medical school?**												.11
	No	52 (7.4)	4 (0.6)	3 (0.4)	12 (1.7)	9 (1.3)	17 (2.4)	7 (1)	0			
	Yes	647 (92.6)	21 (3)	55 (7.9)	81 (11.6)	193 (27.6)	192 (27.5)	100 (14.3)	5 (0.7)			
**Do you use recorded material (lectures or videos) provided by the faculty of medicine at your university?**												.14
	No	198 (28.3)	8 (1.1)	22 (3.2)	30 (4.3)	52 (7.4)	66 (9.4)	21 (3)	0			
	Yes	500 (71.5)	17 (2.4)	36 (5.2)	63 (9)	150 (21.5)	143 (20.5)	86 (12.3)	5 (0.7)			
**Do you use YouTube as a learning tool for pathology?**												.058
	No	124 (17.7)	10 (1.4)	6 (0.9)	17 (2.4)	30 (4.3)	40 (5.7)	20 (2.9)	1 (0.1)			
	Yes	575 (82.3)	15 (2.2)	52 (7.4)	76 (10.9)	172 (24.6)	169 (24.2)	87 (12.5)	4 (0.6)			

^a^N/A: not available.

**Table 3:** The perceived value of using university pathology lectures compared with YouTube lectures (n=699).

Evaluation items				Total sample, n (%)		Score in the pathology course, n (%)															*P* value	
						<50% (n=25)		50%-60% (n=58)		61%-70% (n=93)		71%-80% (n=202)		81%-90% (n=209)		>90% (n=107)		N/A^a^ (n=5)				
**How do you rate the recorded pathology material (lectures or videos) provided by the faculty of medicine at your university?**																						
	**Content**																					.08
		Excellent	60 (8.6)		4 (0.6)		3 (0.4)		6 (0.9)		15 (2.2)		16 (2.3)		16 (2.3)		0					
		Good	233 (33.3)		6 (0.9)		13 (1.9)		27 (3.9)		77 (11)		74 (10.6)		35 (5)		1 (0.1)					
		Acceptable	199 (28.5)		7 (1)		16 (2.3)		28 (4)		63 (9)		57 (8.2)		26 (3.7)		2 (0.3)					
		Poor	145 (20.7)		6 (0.9)		20 (2.9)		24 (3.4)		34 (4.9)		40 (5.7)		20 (2.9)		1 (0.1)					
		Not applicable	62 (8.9)		2 (0.3)		6 (0.9)		8 (1.2)		13 (1.9)		22 (3.2)		10 (1.4)		1 (0.1)					
	**Presentation**																					.03
		Excellent	53 (7.6)		4 (0.6)		3 (0.4)		7 (1)		16 (2.3)		14 (2)		9 (1.3)		0					
		Good	200 (28.6)		3 (0.4)		11 (1.6)		24 (3.4)		58 (8.3)		65 (9.3)		38 (5.4)		1 (0.1)					
		Acceptable	195 (27.9)		9 (1.3)		13 (1.9)		23 (3.3)		69 (9.9)		55 (7.9)		26 (3.7)		0					
		Poor	194 (27.8)		8 (1.1)		23 (3.3)		34 (4.9)		48 (6.9)		53 (7.6)		25 (3.6)		3 (0.4)					
		Not applicable	57 (8.2)		1 (0.1)		8 (1.1)		5 (0.7)		11 (1.6)		22 (3.2)		9 (1.3)		1 (0.1)					
**How do you rate the pathology-related YouTube videos you viewed?**																						
	**Content**																					.02
		Excellent	376 (53.8)		8 (1.1)		26 (3.7)		47 (6.7)		114 (16.3)		115 (16.5)		65 (9.3)		1 (0.1)					
		Good	210 (30)		7 (1)		25 (3.6)		33 (4.7)		58 (8.3)		61 (8.7)		23 (3.3)		3 (0.4)					
		Acceptable	23 (3.3)		2 (0.3)		3 (0.4)		2 (0.3)		10 (1.4)		4 (0.6)		2 (0.3)		0					
		Poor	2 (0.3)		1 (0.1)		0		0		1 (0.1)		0		0		0					
		Not applicable	88 (12.6)		7 (1)		4 (0.6)		11 (1.6)		19 (2.7)		29 (4.2)		17 (2.4)		1 (0.1)					
	**Presentation**																					.01
		Excellent	424 (60.7)		12 (1.7)		28 (4)		56 (8)		125 (17.9)		135 (19.3)		66 (9.4)		2 (0.3)					
		Good	167 (23.9)		5 (0.7)		24 (3.4)		23 (3.3)		48 (6.9)		44 (6.3)		21 (3)		2 (0.3)					
		Acceptable	21 (3)		1 (0.1)		2 (0.3)		3 (0.4)		10 (1.4)		2 (0.3)		3 (0.4)		0					
		Poor	2 (0.3)		2 (0.3)		0		0		0		0		0		0					
		Not applicable	85 (12.2)		5 (0.7)		4 (0.6)		11 (1.6)		19 (2.7)		28 (4)		17 (2.4)		1 (0.1)					

^a^N/A: not available.

**Table 4:** Comparison between attitudes toward the university pathology lectures and YouTube lectures (n=699).

Question	No answer	Disagree	Neutral	Agree
University pathology lectures are not enough.	124 (17.7)	67 (9.6)	185 (26.5)	323 (46.2)
University pathology lectures are not attractive to all students.	124 (17.7)	31 (4.4)	69 (9.9)	475 (68)
YouTube lectures are more interesting.	124 (17.7)	15 (2.2)	76 (10.9)	484 (69.2)
YouTube lectures are more informative.	124 (17.7)	106 (15.2)	180 (25.8)	289 (41.3)
YouTube contains a lot of animations and pictures.	124 (17.7)	6 (0.9)	32 (4.6)	537 (76.8)
YouTube contains many methods of illustrations.	124 (17.7)	2 (0.3)	17 (2.4)	556 (79.5)
YouTube enhances the quality of learning.	124 (17.7)	4 (0.6)	38 (5.4)	533 (76.3)

**Table 5:** Perspective towards the effectiveness of using YouTube as a learning tool for pathology (n=699).

Question		Total sample, n (%)	Score in the pathology course, n (%)								*P* value	
			<50% (n=25)	50%-60% (n=58)	61%-70% (n=93)	71%-80% (n=202)	81%-90% (n=209)	>90% (n=107)	N/A^a^ (n=5)			
**Do you think that using YouTube alone is enough to learn pathology?**												.02
	No	269 (38.5)	12 (1.7)	21 (3)	29 (4.2)	71 (10.2)	85 (12.2)	48 (6.9)	3 (0.4)			
	Maybe	147 (21)	1 (0.1)	12 (1.7)	18 (2.6)	49 (7)	44 (6.3)	22 (3.2)	1 (0.1)			
	Yes	159 (22.8)	2 (0.3)	19 (2.7)	29 (4.2)	52 (7.4)	40 (5.7)	17 (2.4)	0			
**Do you feel confused regarding which pathology content to rely on when using YouTube for learning?**												.13
	No	99 (14.2)	5 (0.7)	9 (1.3)	10 (1.4)	25 (3.6)	31 (4.4)	18 (2.6)	1 (0.1)			
	Maybe	153 (21.9)	2 (0.3)	18 (2.6)	20 (2.9)	48 (6.9)	44 (6.3)	18 (2.6)	3 (0.4)			
	Yes	323 (46.2)	8 (1.1)	25 (3.6)	46 (6.6)	99 (14.2)	94 (13.5)	51 (7.2)	0			
**Do you think that all the available pathology materials corresponded with your learning phase?**												.46
	No	134 (19.2)	6 (0.9)	14 (2)	15 (2.2)	37 (5.3)	40 (5.7)	21 (3)	1 (0.1)			
	Maybe	158 (22.6)	3 (0.4)	15 (2.2)	22 (3.2)	46 (6.6)	50 (7.2)	22 (3.2)	0			
	Yes	282 (40.3)	6 (0.9)	23 (3.3)	39 (5.6)	89 (12.7)	79 (11.3)	43 (6.2)	3 (0.4)			
**Do you think it would be helpful if your instructor chooses the right pathology YouTube videos in his lectures?**												.46
	No	56 (8)	0	4 (0.6)	6 (0.9)	17 (2.4)	20 (2.9)	8 (1.1)	1 (0.1)			
	Maybe	85 (12.2)	3 (0.4)	6 (0.9)	12 (1.7)	23 (3.3)	28 (4)	13 (1.9)	0			
	Yes	434 (62.1)	12 (1.7)	42 (6)	58 (8.3)	132 (18.9)	121 (17.3)	66 (9.4)	3 (0.4)			
**Do you think it would be helpful if your pathology instructor guides you regarding the relevant content to follow on YouTube for the acquisition of knowledge related to a particular topic?**												.048
	No	48 (6.9)	2 (0.3)	5 (0.7)	5 (0.7)	11 (1.6)	11 (1.6)	12 (1.7)	2 (0.3)			
	Maybe	58 (8.3)	1 (0.1)	9 (1.3)	8 (1.1)	16 (2.3)	17 (2.4)	7 (1)	0			
	Yes	469 (67.1)	12 (1.7)	38 (5.4)	63 (9)	145 (20.7)	141 (20.2)	68 (9.7)	2 (0.3)			

^a^N/A: not available.

**Table 6:** Binary logistic regression analysis of studied courses and topics on YouTube and the overall performance of students on pathology tests.

Factor			Students, n (%)		Odds ratio (95% CI)		*P* value
**Pathology sciences and systems studied on YouTube^a^**							
	General pathology	486 (69.5)		0.88 (0.5-1.55)		.67	
	Cardiovascular system	341 (48.8)		1.04 (0.56-1.94)		.89	
	Hematopoietic and lymphoid system	406 (58.1)		0.84 (0.49-1.43)		.52	
	Respiratory system	330 (47.2)		1.65 (0.93-2.94)		.09	
	Central nervous system	249 (35.6)		3.86 (1.33-11.18)		.01	
	Peripheral nervous system	228 (32.6)		0.27 (0.09-0.8)		.02	
	Urogenital system	211 (30.2)		1.06 (0.55-2.06)		.85	
	Gastrointestinal system	267 (38.2)		0.69 (0.38-1.24)		.22	
	Endocrine system	99 (14.2)		0.74 (0.41-1.36)		.34	
**Pathology-related content viewed on YouTube by students^a^**							
	Pathology lectures	502 (71.8)		1.96 (1.08-3.57)		.03	
	Microscopic images	223 (31.9)		1.04 (0.63-1.73)		.87	
	Gross images	270 (38.6)		1.46 (0.88-2.43)		.14	
	Animated pathology videos	402 (57.5)		1.23 (0.79-1.92)		.36	
	Study tips and advice	179 (25.6)		0.47 (0.29-0.77)		<.001	
	Surgical operations	167 (23.9)		1.33 (0.8-2.2)		.28	
	Pathology questions and answers	165 (23.6)		0.94 (0.58-1.54)		.81	
	Others	107 (15.3)		0.76 (0.46-1.27)		.3	
**Percentage of pathology test preparation time spent watching YouTube videos**							
	0%-25%^b^	280 (40.1)		—^c^		.06	
	26%-50%	223 (31.9)		0.58 (0.34-0.99)		.04	
	51%-75%	149 (21.3)		0.46 (0.26-0.82)		.01	
	76%-100%	47 (6.7)		0.51 (0.22-1.2)		.12	
	Constant	—^d^		2.75		.01	

^a^Category (No) was considered the reference category.

^b^Reference category.

^c^Not applicable because it is the reference category.

^d^Not applicable because it is the constant.

## Discussion

### Principal Findings

The findings of our study are in harmony with those of previous studies published in the literature: that YouTube has evolved into one of the most popular social media platforms utilized by many people worldwide for many general purposes and as a learning tool for students in particular [5,13-17]. This was indicated in our study, as 96.5% of the medical students who participated in the survey in Jordan acknowledged using YouTube, and this was reflected in their scores: Very good and good scores were the most frequently described scores (57.9%). There are many published articles about students using YouTube as a learning tool in different specialties [4,6,8,18], with anatomy as a particularly good example [5,13,19,20]. This led us to explore how medical students use YouTube for pathology especially because there have been few previous reports about this topic and because pathology and anatomy share many similarities, particularly in terms of visual engagement [5]. The study showed that 82.2% of the students were using YouTube to learn pathology and that the scores of the students who used YouTube as a tool to learn pathology were higher, with 428 students scoring above 70%. Therefore, based on the results of this study and other studies [5,6,13,18,21], YouTube can be a helpful and preferable educational tool for students, and it should be utilized in a very effective way in universities.

YouTube was used mostly to watch pathology lectures and animated pathology videos. In addition, medical students used it to enhance their understanding of gross and microscopic images. Moreover, students were using YouTube to watch surgical operations to see the pathological organs. These examples were not the only viewed content; students indicated that they were also using YouTube to access study tips and advice videos as well as videos about pathology questions and answers. Further, as in a previous study for anatomy [5], students appear to use YouTube more often to learn about certain topics related to body systems and regions. In our study, students were using YouTube mainly for hematopoietic and lymphoid systems, cardiovascular systems, and other body systems. This might reflect the difficulty of learning these topics and, accordingly, the need to use additional educational resources including YouTube videos. It is worth mentioning that watching YouTube videos to study the central nervous system was associated with higher odds of achieving higher scores but was related to lower odds when studying the peripheral nervous system. This could be attributed to the difficulty of the content in the peripheral nervous system course, the difficulty in memorizing the details of nerves and their distribution, and the possible poor quality of YouTube videos. Surprisingly, spending more time watching pathology videos on YouTube while studying for examinations corresponded with lower performance, and this might be explained by distraction caused by watching many videos, focusing on materials not related to their curricula, watching without memorization, and loss of concentration.

Medical students seem to have a very positive attitude toward YouTube as a pathology learning tool. Almost all the students agreed that they found useful pathology-related information on YouTube and that YouTube helped understand pathology topics, memorize and recall pathological information, and achieve higher scores in pathology-related exams. This combination of memorization, understanding, and visualization is needed for successful learning in most courses, especially those including pictures and images such as anatomy and pathology in medical schools [5,22]. Actually, videos are suggested to have a positive impact on these mental processes. This is fortified by the results of a study in which videos uploaded to an anatomy YouTube channel were reported to be helpful in creating memorable visual images [13]. Moreover, most of the students in our study encouraged their faculty members to make their own pathology YouTube channels to be a reference for them and their colleagues in the future.

### Comparison With Prior Work

The content and presentation of pathology-related YouTube videos were found to be superior to those of recorded pathology lectures or videos. This was mainly attributed to including animations and pictures and using a variety of methods for illustrations. Moreover, medical students perceived pathology lectures on YouTube as more attractive, informative, and interesting, and they enhanced the quality of pathology learning, which is consistent with many previous studies [4,5,13-17]. This indicates the importance of the available online platforms including YouTube as a supplementary educational tool. However, it should be noted that, although YouTube can offer educational value, it should not be solely relied upon for pathology learning, passing pathology exams, and obtaining the needed information for the curricula, as supported by many students in our study and a previous study on embryology and histology [13] that found that 34.2% and 25% of students were hesitant to use YouTube to learn embryology and histology, respectively. This suggests that labeled images, true histopathological slides, and virtual microscopy are preferred over videos.

Moreover, although the recorded material (lectures or videos) provided by the faculty of medicine at their universities was the least often mentioned source of information (198/699, 28.3%), students who relied only on this material stilled received very good and good scores. This suggests that pathology educators could benefit from creating their own YouTube channels and incorporating them into their face-to-face lectures, as this approach has been found to improve the learning process [5,23].

It is worth noting that YouTube videos are of variable quality and are, accordingly, of varying educational value, which might be challenging and time-consuming for students, as indicated in our study and another corresponding study about anatomy [5]. Students were confused regarding which content to rely on when using YouTube for learning, and the available content-related material or course did not correspond with their learning phase, despite achieving good or very good scores. On the other hand, most of the students who claimed that they were not confused when selecting the appropriate pathologic information achieved a score higher than 70%. As explored in our study, this could necessitate the help of instructors by using some YouTube videos in their lectures, guiding the students on the relevant pathology content to follow on YouTube, and providing them with suggested links that meet the course objectives and match the student’s level of knowledge.

Overall, this study adds to the existing literature on the potential use of YouTube as an effective supplementary pathology learning tool. It also emphasizes the importance of using a variety of methods and sources to ensure comprehensive education in pathology.

### Strengths, Limitations, and Future Directions

#### Recommendations

In light of this, pathology educators at Jordanian universities may consider the potential benefits of using YouTube as an educational tool. This could be achieved by providing their students with educationally effective pathology channels while also admitting the possible limitations and drawbacks of this approach.

Moreover, pathology educators could consider the creation of a pathology YouTube channel for Jordanian universities that prompt discussion and sharing of information while ensuring students’ privacy and academic integrity.

#### Limitations

Although this study has provided explorative information about the use of YouTube to learn pathology, it has some limitations. First, the questionnaire was general and did not include questions that evaluated students’ satisfaction with specific YouTube channels that are frequently used by the students. Future research could consider using a mixed methods approach that includes both self-reported data and objective measures to investigate this issue in addition to investigating the usefulness of creating new YouTube channels for Jordanian universities. Second, although the study included an adequate sample size, generalization of the results must not be easily assumed, since some universities had much greater participation than others (nearly 50% of the participants were from Yarmouk University), and some groups had higher participation rates (second-year students). Future studies could consider recruiting students with near equal proportion from different universities at different levels of medical study. Third, this study only focused on the use of YouTube as a supplementary learning tool, and other aspects such as the videos’ quality, level of engagement, or learning preferences of the students were not investigated. Future research could explore these factors to provide a more comprehensive understanding of the use of YouTube as a learning tool. Fourth, since we conducted our study amid the COVID-19 era, not considering the effect of the COVID-19 pandemic on students' education is considered another limitation of this study. Therefore, we recommend other investigators study the effect of COVID-19 lockdowns on the online learning process in general and using YouTube channels in particular. Last, this study did not address any limitations pertaining to copyright laws or other ethical issues associated with using open-source content on YouTube. Future studies could also consider addressing these ethical considerations to ensure that utilizing YouTube as a resource for learning is legally and ethically sound.

### Conclusion

The results of this study suggest that YouTube may play a role in enhancing pathology learning, and aiding in understanding, memorization, recalling information, and obtaining higher scores. Many medical students in Jordan have positive attitudes toward using YouTube as a supplementary pathology learning tool. Based on this, it is recommended that pathology instructors should explore the use of YouTube and other emerging educational tools as a potential supplementary learning resource.

## Abbreviations

CHERRIES: Checklist for Reporting Results of Internet E-Surveys
OR: odds ratio

## Data Availability

The data sets generated during and/or analyzed during the current study are available from the corresponding author upon reasonable request.

## References

[1] Posts tagged digital 2021. DataReportal.

[2] (2010). YouTube EDU Turns One Today. Open Culture.

[3] Buzzetto-More N (2015). Student attitudes towards the integration of YouTube in online, hybrid, and web-assisted courses: an examination of the impact of course modality on perception. Journal of Online Learning & Teaching.

[4] A. Buzzetto-More N (2014). An examination of undergraduate student’s perceptions and predilections of the use of YouTube in the teaching and learning process. IJELL.

[5] Mustafa A, Taha N, Alshboul O, Alsalem M, Malki M (2020). Using YouTube to learn anatomy: perspectives of Jordanian medical students. Biomed Res Int.

[6] George D, Dellasega C (2011). Use of social media in graduate-level medical humanities education: Two pilot studies from Penn State College of Medicine. Medical Teacher.

[7] Patel S, Mauro D, Fenn J, Sharkey D, Jones C (2015). Is dissection the only way to learn anatomy? Thoughts from students at a non-dissecting based medical school. Perspect Med Educ.

[8] Tan E, Pearce N (2011). Open education videos in the classroom: exploring the opportunities and barriers to the use of YouTube in teaching introductory sociology. Research in Learning Technology.

[9] Eysenbach G (2012). Correction: improving the quality of web surveys: the Checklist for Reporting Results of Internet E-Surveys (CHERRIES). J Med Internet Res.

[10] Dean A, Arner T, Sunki G, Friedman R, Lantinga M, Sangam S, Zubieta J, Sullivan K, Brendel K, Gao Z, Fontaine N, Shu M, Fuller G, Smith D, Nitschke D, Fagan R (2011). Epi Info. Centers for Disease Control and Prevention.

[11] Seetan K, Al-Zubi M, Rubbai Y, Athamneh M, Khamees A, Radaideh T (2021). Impact of COVID-19 on medical students' mental wellbeing in Jordan. PLoS One.

[12] WMA Declaration of Helsinki - Ethical principles for medical research involving human subjects. World Medical Association.

[13] Jaffar A (2012). YouTube: An emerging tool in anatomy education. Anat Sci Educ.

[14] Snelson C (2011). YouTube across the disciplines: a review of the literature. Journal of Online Learning and Teaching.

[15] Cha M, Kwak H, Rodriguez P, Ahn YY, Moon S (2007). I tube, you tube, everybody tubes: analyzing the world's largest user generated content video system. IMC '07: Proceedings of the 7th ACM SIGCOMM conference on Internet measurement.

[16] Gill M, Arlitt M, Li Z, Mahanti A (2007). Youtube traffic characterization: a view from the edge. IMC '07: Proceedings of the 7th ACM SIGCOMM conference on Internet measurement.

[17] Rotman D, Preece J (2010). The 'WeTube' in YouTube - creating an online community through video sharing. IJWBC.

[18] Chtouki H, Harroud H, Khalidi M, Bennani S (2012). The impact of YouTube videos on the student's learning.

[19] Barry D, Marzouk F, Chulak-Oglu K, Bennett D, Tierney P, O'Keeffe GW (2016). Anatomy education for the YouTube generation. Anat Sci Educ.

[20] Reverón RR (2016). The use of YouTube in learning human anatomy by Venezuelan medical students. MOJ Anatomy & Physiology.

[21] Berk RA (2009). Teaching strategies for the net generation. Transformative Dialogues: Teaching & Learning Journal.

[22] Pandey P, Zimitat C (2007). Medical students' learning of anatomy: memorisation, understanding and visualisation. Med Educ.

[23] Sanchez-Diaz PC (2013). Impact of interactive instructional tools in gross anatomy for optometry students: a pilot study. Optometric Education.

